# Free-Standing 3D Printing of Epoxy-Vinyl Ether Structures Using Radical-Induced Cationic Frontal Polymerization

**DOI:** 10.1021/acsapm.3c02226

**Published:** 2023-12-15

**Authors:** Brecklyn R. Groce, Alexandra V. Aucoin, Md Asmat Ullah, Jake DiCesare, Claire Wingfield, Jonathan Sardin, Jackson T. Harris, John C. Nguyen, Patrick Raley, Svetlana S. Stanley, Genevieve Palardy, John A. Pojman

**Affiliations:** † Department of Chemistry, Louisiana State University, Baton Rouge, Louisiana 70803, United States; ‡ Department of Mechanical and Industrial Engineering, Louisiana State University, 3261 Patrick F. Taylor Hall, Baton Rouge, Louisiana 70803, United States

**Keywords:** frontal polymerization, additive manufacturing, cationic polymerization, epoxy, vinyl ether, carbon fiber, carbon nanofiber

## Abstract

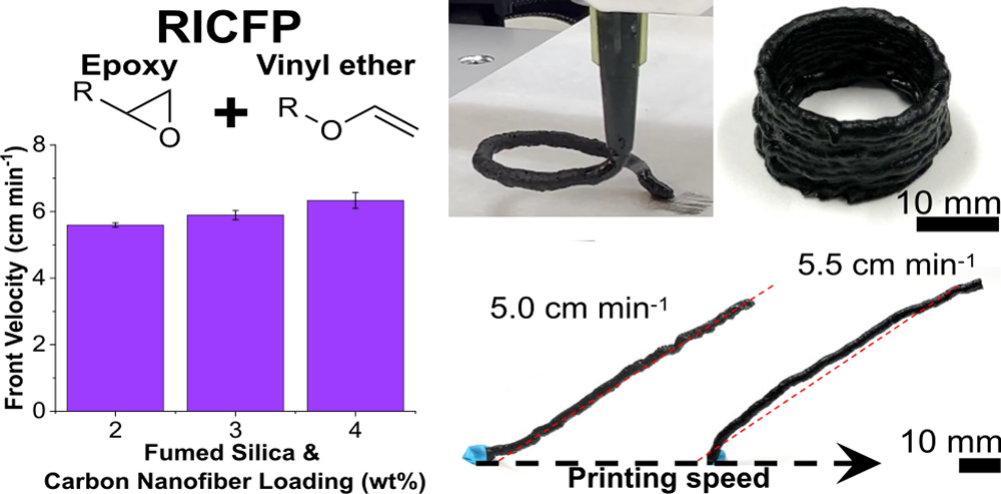

The application of frontal polymerization to additive manufacturing has advantages in energy consumption and speed of printing. Additionally, with frontal polymerization, it is possible to print free-standing structures that require no supports. A resin was developed using a mixture of epoxies and vinyl ether with an iodonium salt and peroxide initiating system that frontally polymerizes through radical-induced cationic frontal polymerization. The formulation, which was optimized for reactivity, physical properties, and rheology, allowed the printing of free-standing structures. Increasing ratios of vinyl ether and reactive cycloaliphatic epoxide were found to increase the front velocity. Addition of carbon nanofibers increased the front velocity more than the addition of milled carbon fibers. The resin filled with carbon nanofibers and fumed silica exhibited shear-thinning behavior and was suitable for extrusion-based printing at a weight fraction of 4 wt %. A desktop 3D printer was modified to control resin extrusion and deposition with a digital syringe dispenser. Flexural properties of molded and 3D-printed specimens showed that specimens printed in the transverse direction exhibited the lowest strength, likely due to the presence of voids, adhesion issues between filaments, and preferential carbon nanofiber alignment along the filaments. Finally, free-standing printing of single, angled filaments and helical geometries was successfully demonstrated by coordinating ultraviolet-based reaction initiation, low air pressure for resin extrusion, and printing speed to match front velocity.

## 1 Introduction

Frontal polymerization (FP) is a process wherein an initial stimulus initiates a localized polymerization reaction that propagates through uncured resin, curing the resin as it travels. The process relies on heat diffusion and the Arrhenius rate kinetics of an exothermic reaction.^1−4^ FP was used with various monomer systems that have different mechanisms of polymerization, such as acrylates that use free-radical polymerization,^5−7^ dicyclopentadiene using ring-opening metathesis polymerization,^8−10^ and urethane polymerizations mediated by catalysts.^11,12^

Radical-induced cationic frontal polymerization (RICFP) allows for FP of epoxies and vinyl ethers through the combination of a thermal radical initiator that promotes decomposition of a superacid-generating salt.^13−15^ It can be initiated by either heat or light. Typically, iodonium-based salts are used in RICFP as the superacid-generating salts, with hexafluoroantimonate or aluminate counterions being common. Peroxides^14^ and benzopinacol, which is a gas-less initiator,^16^ are common in RICFP. During RICFP, if thermally initiated, the radical initiator decomposes to produce radicals that reduce the superacid generator, which generates a superacid based on the counterion after decomposition steps. The superacid can then initiate cationic polymerization, and heat from propagation generates radicals from the radical initiator, looping the process.^14,17^ If initiated by ultraviolet (UV) light, the superacid generator instead decomposes and generates the superacid from excitation by the UV, which then initiates polymerization, and the heat generated by propagation cleaves the radical initiator to promote superacid generator decomposition.^13^ A simplified mechanism of the RICFP process is shown in Figure S1.

Additive manufacturing (AM or 3D printing) using FP has been explored and reported in the literature. Frontal ring-opening metathesis polymerization has been used extensively to demonstrate the potential of 3D printing through FP.^18−21^ The use of FP for 3D printing has advantages in energy consumption and the speed of printing. An ideal process will see that the front is initiated and propagates behind the extruded material very closely so that the resin does not sag, owing to suitable viscosity, and the front is continuous.

There are a few reports of RICFP being used for 3D printing. In their first publication on the topic, Zhang et al.^22^ investigated printing a formulation containing a commercial bisphenol A diglycidyl ether (BADGE)-based epoxy resin with an iodonium aluminate salt and benzopinacol initiating system. They soaked continuous carbon fiber (CF) tows with the formulation and successfully cured this material frontally while extruding, with an increase in front velocity when the resin was soaked into carbon fibers. A subsequent report by Zhang et al.,^23^ using the same formulation as above, saw that front velocity increased with the addition of 1 wt % carbon nanotubes (CNTs) and formulations with the CNTs and continuous CF tows resulted in improved mechanical properties. CNTs are fillers with high thermal diffusion and have a much smaller diameter than carbon fibers with a higher specific surface area.^24^ Zhang et al.^25^ performed a detailed study of the effects of CNTs, graphene oxide, and discontinuous CFs on RICFP for printing. Using the same resin, they found that 1 wt % CNTs or 1 wt % discontinuous CFs gave small increases in front velocity compared to the neat resin, while 1 wt % graphene oxide reduced the front velocity. They also demonstrated the printing of a spiral shape using epoxy resin with CNTs. In the three papers above, a heat bed set to 120 °C was used to initiate the fronts. Gao et al.^26^ investigated the frontal curing of a printed highly viscous novolac epoxy resin with iodonium aluminate salt and benzopinacol, through spraying of the initiating system with an atomizer while printing. This was initiated with a 90 °C heat bed, and a dependence of front velocity was seen with layer thickness and atomizer parameters.

In this paper, we intend to demonstrate the potential of printing free-standing structures using the frontal polymerization of epoxy-vinyl ether composites. Vinyl ethers were previously shown to increase reactivity when added to epoxy systems.^14^ By using a vinyl ether in tandem with two different epoxies, we aim to formulate a reactive system with desirable rheological properties for extrusion-based 3D printing. The effects of resin composition, initiator concentration, filler type, and loading on front kinetics were investigated. Carbon nanofibers (CNFs) and milled carbon fibers (MCFs) were compared as fillers to tune front kinetics, and CNFs were investigated to manipulate mechanical properties, while fumed silica (FS) was used to further tailor viscosity, as FS is inert in RICFP.^14,17^ CNFs are smaller than carbon fibers and have a higher specific surface area, which could assist in improving physical properties. They are larger than CNTs, less thermally conductive, and differ in their physical structure, being cylinders composed of stacked layers versus hollow CNTs;^24,27^ CNFs have the advantage of being less expensive than CNTs while still possessing beneficial thermal properties for FP. Printing parameters of the resin were investigated, along with the rheological behavior and mechanical and microscopic analysis of the generated composites. Finally, we demonstrated the ability to print free-standing structures with this filled resin.

## 2 Materials and Methods

### 2.1 Materials

**Figure 1:**
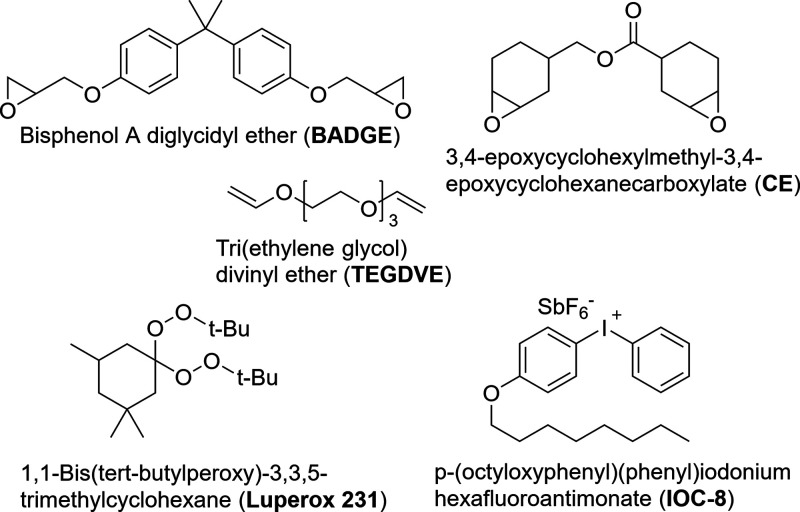
Structures of monomers and initiators used in printing resin.

2,2-Bis(4-glycidyloxyphenyl)propane (bisphenol A diglycidyl ether, BADGE) was obtained from TCI Chemicals (Montgomeryville, PA). 3,4-Epoxycyclohexylmethyl-3,4-epoxycyclohexanecarboxylate (CE) was purchased from Ambeed Inc. (Arlington Heights, IL). Tri(ethylene glycol) divinyl ether (TEGDVE) and 1,1-Bis(tert-butylperoxy)-3,3,5-trimethylcyclohexane (Luperox 231) were purchased from Sigma Aldrich (St. Louis, MO), and *p*-(octyloxyphenyl)(phenyl)iodonium hexafluoroantimonate (IOC-8) was purchased from Hampford Research Inc. (Stratford, CT). Aerosil 200 FS was obtained from Evonik (Piscataway, NJ). Zoltek PX35 milled carbon fibers (MCFs) were purchased from Zoltek Companies, Inc. (St. Louis, MO), and PR-19-XT-HHT carbon nanofibers (CNFs) were purchased from Pyrograf Inc. (Cedarville, OH). All chemicals were used as received, and their structures are shown below in Figure 1.

### 2.2 Preparation of Formulations and Front Velocity Measurements

The resin was comprised of a mixture of 60 wt % BADGE, 20 wt % CE, and 20 wt % TEGDVE. To prepare formulations for frontal polymerization, first, IOC-8 was dissolved in a mixture of BADGE and CE using a heated sonicator at approximately 40 °C. After dissolution, TEGDVE and Luperox 231 were added, and the mixture was stirred for 10 min using a high shear mixer with a propeller. The IOC-8 acts as a superacid generator, while Luperox 231 acts as a thermal radical initiator that produces radicals that induce the decomposition of the IOC-8. For the fillers, FS was added and then the formulation was mixed in a FlackTek speed mixer at 800 rpm for 2 min. Then, either MCFs or CNFs were added, and the formulation was mixed once at 900 rpm for 1 min, followed by two cycles at 1800 rpm for 3 min.

For front kinetic measurements, the formulation was loaded into a wooden mold (135 × 20 × 6 mm) lined with wax paper. Polymerization was initiated by contact using a soldering iron heated to 200 °C. The front was observed with a video camera, and the front velocity was calculated from the slope of the front position versus time. Where indicated, the formulation was instead spread onto a piece of plywood covered in wax paper at a thickness of 1.5 mm by using a drawdown bar. The thickness of 1.5 mm was selected because it was closer to the dimension of a printed filament.

The degree of cure of frontally polymerized materials was also determined by using a differential scanning calorimeter (DSC Q100, TA Instruments, New Castle, DE). A ramp rate procedure from 0 to 250 °C at 10 °C min^–1^ was used for both the uncured resin and cured polymer. The degree of cure was calculated by dividing the area of the residual exothermic peak of the cured polymer by the area of the exothermic peak of curing for the uncured resin.

### 2.3 Rheological Characterization

A parallel plate Discovery Hybrid Rheometer 20 (DHR-20, Waters TA Instruments) was employed to measure shear viscosity for resin formulations containing different FS and CNF weight fractions (0 to 6 wt %). Resin samples were tested at 25 °C with 25 mm diameter parallel plates in shear rate sweep mode from 0.1 to 100 s^–1^. This data was used to study the shear-thinning behavior of the polymer and establish which resin formulations would be suitable for free-standing 3D printing at room temperature. The main goal was to achieve a viscosity allowing consistent extrusion through the extruder nozzle while avoiding sagging of the extruded material at the nozzle tip.

### 2.4 Morphological Characterization

After polymerization, as described in Section 2.2, samples were manually fractured, and the surfaces were observed by scanning electron microscopy (SEM) to assess the presence of voids and the dispersion state of the carbon nanofibers or milled carbon fibers. The SEM images were taken with a high-performance JSM-6610LV SEM instrument with a voltage of 15 kV. SEM imaging of milled carbon fiber composites was performed with the Thermo Scientific Helios G4 PFIB CXe at a voltage of 5 kV. Before SEM, the fractured samples were spray-coated with gold in a sputter coater (EMS550X) at 25 mA and a vacuum of 1 × 10^–1^ mbar for 2 min.

The void content of the polymers produced by frontal polymerization was estimated by gravimetric density measurements based on ASTM D2734. Sample volume was limited to less than 2 cm^3^ contrary to that written in the standard method, and the theoretical density of the polymer could not be calculated as indicated in the standard method but was instead measured empirically. These limitations arise from the porous nature of the system. A polymer sample with minimal voids to serve as a reference density was cured in an oven at 100 °C. The density of samples without voids and polymer samples made by frontal polymerization was determined by dividing the sample weight by the volume, which was found by measuring each side of the sample. Void content, *V*, was calculated using eq 1 below, derived from ASTM D2734, where ρ_r_ is the density of the reference with no voids and ρ_s_ is the density of the sample. Samples were cut to size by using a small table saw.
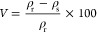
1

### 2.5 Extrusion-Based Additive Manufacturing

**Figure 2:**
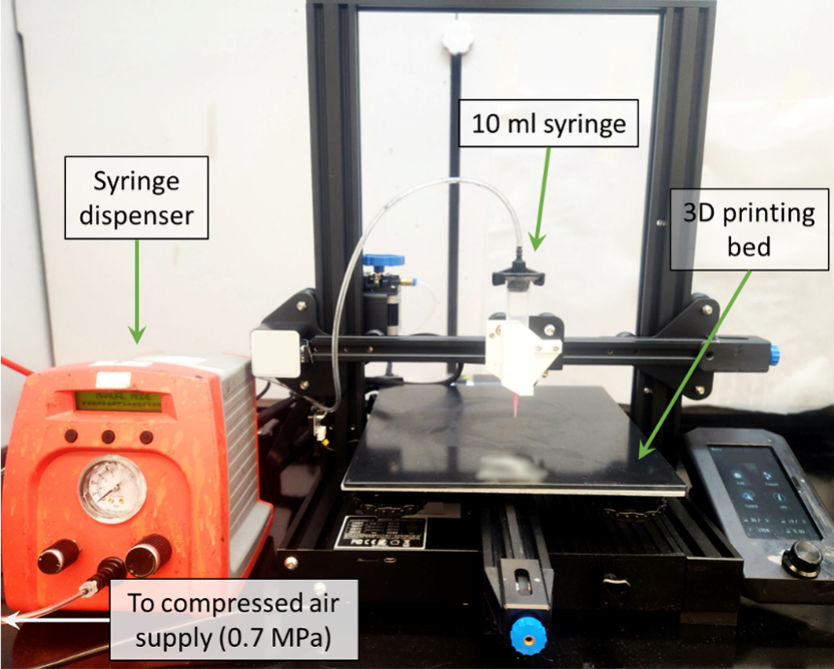
Modified 3D printing setup for small-scale study with FP resin. A syringe dispenser was used to control the flow of the resin from a 10 mL syringe.

Two extrusion-based AM setups were considered for this study. A robotic setup with a UR5 manipulator was first employed to assess the feasibility of layer-by-layer 3D printing with the FP resin system and identify the main issues. Cylindrical geometries were printed based on a computer-aided design (CAD) model with an outer diameter of 20 mm, a height of 12 mm, and a wall width of 1.5 mm. The setup and manufacturing process were described in detail elsewhere.^28,29^ To further investigate the behavior of the FP resin for small-scale, free-standing extrusion-based AM, a desktop 3D printer was then modified and used as the main AM setup in this study (Figure 2).

This was achieved by using an Ender-3 V2 3D printer and replacing its heated fused deposition extruding unit with a custom-made syringe holder. The latter was 3D-printed with polylactic acid (PLA) and designed to hold a 10 mL syringe as an extruder. To obtain material flow, a tubing system was employed, connecting the syringe to a pressure controller. For regulating the pressure, a syringe dispenser (LOCTITE digital syringe dispenser, Henkel, Rocky Hill, CT) was utilized, able to adjust pressure within a range of 0 to 0.7 MPa (0 to 7 bar). A nozzle was attached at the tip of the syringe, and its inner diameter measured 2 mm, unless specified otherwise. The printing platform maintained an average temperature of 25 °C. The process involved depositing the FP resin onto the platform, followed by quick thermal initiation. Two initiation methods were compared: (1) soldering iron heated to 200 °C and (2) two SkyBeam UV spotlights at 100% intensity (10 W, 365 nm wavelength, 6 mm lens, 5.6 W cm^–2^ at a distance of 13 mm, UVitron International, West Springfield, MA).

The modified 3D printing setup was used to study the extrusion behavior of the FP resin for different filler weight fractions (FS and CNFs) under different pressures (0.02 to 0.15 MPa), nozzle diameters (1.5 and 2.0 mm), and printing speeds (1.5 to 6 cm min^–1^ or 0.25 to 1.0 mm s^–1^). Videos of the extrusion at the nozzle were captured, and deposited filament width and thickness were measured with a caliper to find a suitable set of parameters based on resin formulation. The main goal was to find filler weight fraction, pressure, diameter, and printing speed combinations to achieve consistent material extrusion while avoiding material sagging at the nozzle exit to enable free-standing printing. Once a formulation was selected, planar specimens were printed for mechanical characterization (Section 2.6). Free-standing printing was then demonstrated with single filaments printed at an angle (40°) and with helical geometries. Sample geometries were modeled in SolidWorks, then imported in UltiMaker Cura 5.4 (Netherlands) as .stl files and saved as .gcode files for the printing process. For free-standing printing, gcode files were manually modified to produce single paths.

### 2.6 Mechanical Performance Characterization

**Figure 3:**
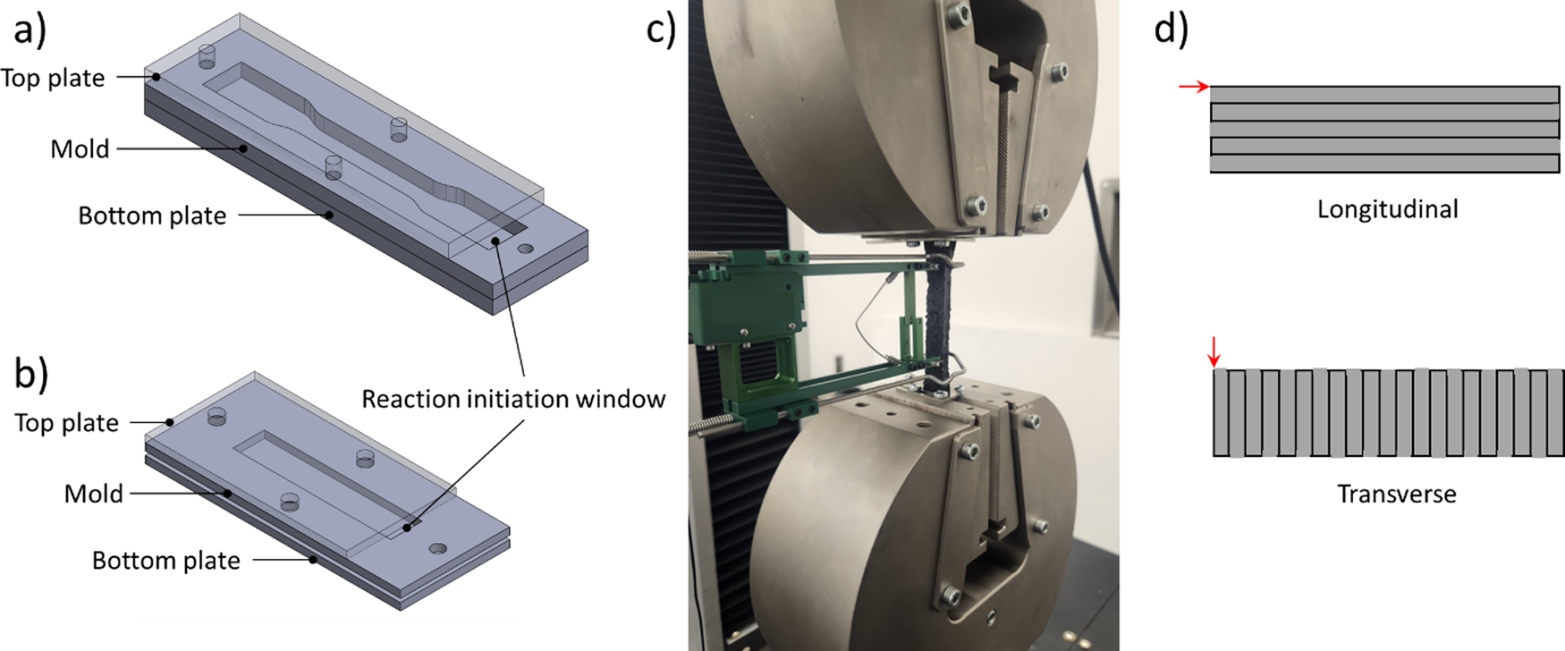
Mold geometries for (a) tensile test specimens and (b) 3PB specimens. (c) Molded dogbone specimen during tensile testing and (d) 3D-printed specimens for 3PB in the longitudinal and transverse directions. 3D models in (a) and (b) are not to scale.

Tensile tests were performed with a 50 kN test machine (TestResources 313) on molded specimens for different FP resin formulations to assess the effect of the filler content (FS and carbon nanofibers). Dogbone specimens were molded with a 3-part acrylic mold based on ASTM D638 Type I geometry, as shown in Figure 3a. It was coated with a release agent; then the resin was poured into the dogbone mold and pressed with a top acrylic plate, and the reaction was started with a soldering iron at a temperature of 200 °C at one end of the sample. The specimens were lightly sanded before testing to remove sharp edges and surface defects. For tensile testing, the specimens were clamped with hydraulic grips, and an extensometer (E3442, 50.8 mm gage, Epsilon Technology Corp., Jackson, WY) was positioned on each sample to acquire displacement data under a loading rate of 1.3 mm min^–1^ (Figure 3c). Each experiment was carried out on six to eight molded specimens (*n* = 6 to 8) for each resin formulation. Ultimate strength, elastic modulus, and strain at break were obtained from the stress–strain curves as well as their corresponding standard deviations. To remove any outliers, Chauvenet’s Criterion was used when analyzing all data.

To compare mechanical performance between molded and 3D-printed specimens, a rectangular three-point bending (3PB) geometry was employed based on ASTM D790. It allowed 3D printing of specimens in the longitudinal and transverse directions (shown in Figure 3d) to evaluate the effect of filament orientation on mechanical performance under bending. Rectangular specimens were molded with a three-part acrylic mold as shown in Figure 3b. It was coated with a release agent, then the resin was poured into the mold, pressed with a top acrylic plate, and the reaction was started with a soldering iron at a temperature of 200 °C at one end of the sample. Rectangular specimens had a base length of 65 mm, a width of 12.5 mm, and a thickness of 5 mm. Both molded and 3D-printed specimens were lightly sanded before testing to remove sharp edges or surface defects. A TestResources 50 kN test machine, equipped with a 3PB fixture, was employed for flexural loading at a rate of 1.3 mm min^–1^ until failure. The supports were placed symmetrically beneath the rectangular specimens with a span of 47 mm. During testing, load-displacement curves were acquired, and the flexural strain (ϵ) and stress (σ) were calculated with eqs 2 and 3, respectively:
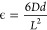
2
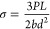
3where *D* is the cross-head displacement (mm), *d* is the specimen’s thickness (mm), *L* is the span length (mm), *P* is the applied load (N), and *b* is the specimen’s width (mm). Each experiment was carried out on six to eight specimens (*n* = 6 to 8) for each molded and printed geometry. To remove any outliers, the Chauvenet’s Criterion was used when analyzing all data.

## 3 Results and Discussion

### 3.1 Comparison of Composite Formulations

#### 3.1.1 Front Velocity

The resin formulation was chosen as it resulted in rigid BADGE-based polymers, with dilution of the viscous BADGE to a workable viscosity by CE and TEGDVE, as they are both reactive monomers that increase front velocity compared to a formulation containing only BADGE.^14,30^ Since the printing process is extrusion-based and extrusion speed may be limited by how fast the front can propagate, especially for free-standing printing, maximizing the front velocity while maintaining desirable physical properties is optimal. It was first found that the 3:1 BADGE:TEGDVE (wt:wt) with either 1 phr IOC-8 or 2 phr IOC-8 and 1 phr Luperox 231 formulations would support fronts in layers as thick as the wooden mold described in Section 2.2, and produce rigid and strong polymers. However, at thinner diameters closer to the printing diameter, the front would be quenched. Increasing the TEGDVE to a 1:1 BADGE:TEGDVE (wt:wt) ratio with 2 phr IOC-8 and 1 phr Luperox 231 solved the reactivity issue, but the rigidity of the polymer appeared to decrease. This is likely due to the differences in structure of the TEGDVE versus BADGE, where the former is a structurally linear monomer with ether linkages that facilitate bending compared to the aromatic rings of BADGE, which provide rigidity. Polymers containing TEGDVE with epoxy and produced with RICFP have been previously reported to be flexible.^14,17^ To maintain reactivity but improve the rigidity of the polymer, CE was added, which contains two cyclohexane rings for increased rigidity unlike TEGDVE. The final resin composition of 3:1:1 BADGE:CE:TEGDVE (wt:wt:wt) with 1 phr IOC-8 and 1 phr Luperox 231 was chosen as the optimal balance of reactivity and physical properties while supporting a front at the printing diameter to allow for the front-driven printing.

In the literature, systems of pure BADGE, 1 mol % IOC-8, and 1 mol % benzopinacol have been found to have a front velocity of 2.7 cm min^–1^.^16^ Notably, these systems are also found to not support fronts below 1 mol % IOC-8. The front velocities we report here by adding TEGDVE and CE to BADGE are higher than these literature results and support fronts at lower concentrations of IOC-8; only 0.44 mol % IOC-8 (equivalent to 1 phr IOC-8) was needed to support a front with the 3:1:1 BADGE:CE:TEGDVE system. When comparing systems containing reactive diluents like the TEGDVE and CE, front velocities are comparable to the literature where the velocity ranges from 4.6 to 4.8 cm min^–1^ with CE and 1,4-butanediol diglycidyl ether added to BADGE with an IOC-8 and benzopinacol initiating system.^30^ The lower minimum IOC-8 concentration is advantageous for lessened material requirements, and the greater velocity of the resin with TEGDVE and CE added is beneficial to the print speed.

**Figure 4:**
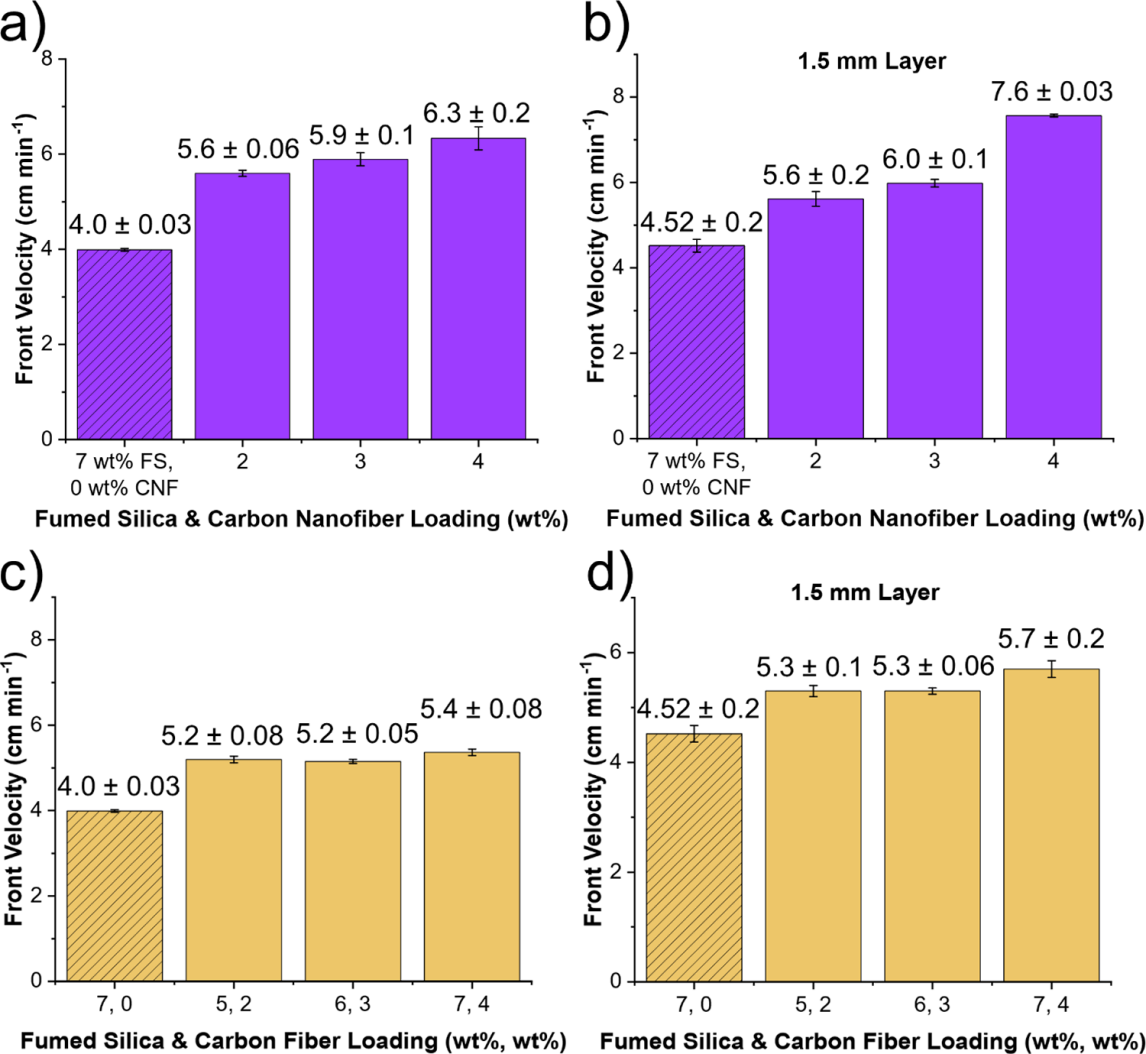
Comparison of front velocity versus filler loading for samples with the printing resin. (a) Front velocity versus fumed silica (FS) and carbon nanofiber (CNF) loading, where equivalent wt % of each filler was added. (b) Front velocity versus FS and CNF loading in a 1.5 mm layer, where equivalent wt % of each filler was added. (c) Front velocity versus FS and milled carbon fiber loading, where the wt % of each filler is shown separated respectively by commas. (d) Front velocity versus FS and milled carbon fiber loading in a 1.5 mm layer, where the wt % of each filler is shown separated respectively by commas. Data for the resin with only 7 wt % FS added are shown in each graph and designated by dashed lines in the bar.

Carbon-based fillers were assessed as a means of increasing viscosity so that extrusion could continue without sagging of the material at the nozzle tip while also affecting the front kinetics and allowing for a faster printing process. An increasing amount of both FS and CNFs from 2 to 4 wt % added to the resin was found to result in an increase in front velocity, as shown in Figure 4. With milled carbon fibers, however, the front velocity only increased a small amount at the highest loading studied of 7 wt % FS and 4 wt % MCF. The nonequivalent loadings of FS and carbon fiber are a result of qualitatively matching the viscosity of the FS and carbon nanofiber resin. The increasing front velocity was not unexpected based on previous publications regarding the addition of conductive elements to frontally polymerized resins.^7,17,31,32^ However, the addition of conductive fillers, such as carbon nanofibers or carbon fibers, aids in heat diffusion. Testing the resin with only FS and no carbon fillers resulted in a lower front velocity of 4.0 cm min^–1^, indicating that the addition of carbon filler is aiding in heat diffusion and subsequent increase of front velocity.

The degree of cure for 4 wt % FS and 4 wt % CNF or 7 wt % FS and 4 wt % MCF samples was also assessed by differential scanning calorimetry, where a high average degree of cure of 98.5% was found for the CNF sample. For the MCF sample, a degree of cure of 99.2% was found. A representative curve from each sample is shown in Figures S2 and S4 in Supporting Information. Detailed curves of the cured CNF and MCF samples are presented in Figures S3 and S5, respectively.

For milled carbon fibers, there are possible factors that may cause the much smaller increase in front velocity compared with carbon nanofibers. First, the higher surface area of carbon nanofibers could impact the front velocity more, due to interactions with the system. A higher loading of either conductive element is likely to increase front velocity up to a maximum loading when the front velocity suffers due to heat loss to the excess filler. Previous additions of conductive fillers to FP resins in the literature used approximately 30 wt % milled carbon fiber to result in an increase in front velocity for RICFP systems,^17^ or 49 wt % milled carbon fiber for free-radical acrylate FP.^7^ Both of these previous literature reports use a much higher mass of carbon fillers than that of the printing formulations shown here. Studies of other carbon fiber composites have used plies of woven carbon fibers to witness an increase in front velocity due to thermal conductivity.^33,34^ The thermal conductivity of milled carbon fibers is lower, 6.4 W m^–1^ K^–1^,^7^ than carbon nanofibers, 1950 W m^–1^ K^–1^.^24^ It is unlikely that the FS addition is affecting the front velocity as previous reports show front kinetics are unaffected above a critical minimum viscosity to overcome convective effects.^17,35,36^

It was also found that front velocity would increase with filler loading at a 1.5 mm thick layer, as shown in Figure 4b,d. The wooden mold used in the previous experiments has a thickness of 6 mm, which is much thicker than the actual diameter of the resin when it is extruded from the printer. Thin layers in frontal polymerization suffer from higher heat loss than thicker samples due to the increased surface area to volume ratio, which can typically quench or slow fronts.^1,2,35^

Surprisingly, the front velocity was slightly higher at 1.5 mm thick layers than at the 6 mm thick layers as filler content increased, especially for the most filled systems. The trend occurred for both milled carbon fiber and carbon nanofibers. This is contrary to the previous reports in the literature, which would indicate that the front velocity is lower in thinner layers due to an increase in surface area to volume ratio that results in heat loss in the system.^35,37,38^ The cause of this anomalous result cannot be explained by previous reports and requires further investigation.

#### 3.1.2 Rheological Behavior

**Figure 5:**
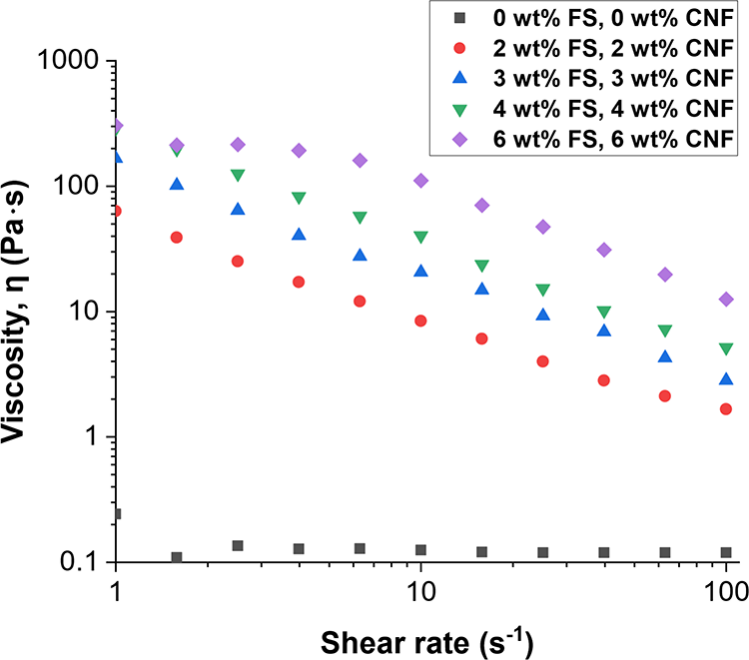
Viscosity profiles from steady-state shear experiments showing the effect of the shear rate on the rheological behavior of the printing resin containing different weight fractions of fumed silica (FS) and carbon nanofiber (CNF).

A study of the rheological properties of the printing resin containing different loadings of FS and carbon nanofiber was also performed. An expected increase in viscosity was seen with increasing FS and carbon nanofibers in the viscosity profiles presented in Figure 5. The viscosity decreased with increasing shear rate, indicating that the filled resin possesses shear-thinning behavior, which is beneficial to extrusion-based 3D printing. Similar FS-filled epoxy resins meant for frontal polymerization were found to exhibit shear-thinning behavior, where viscosity decreased with an increase in shear strain or shear rate.^17^ The unfilled printing resin did not appear to exhibit the same behavior. Instead, its viscosity remained relatively constant when the shear rate increased. Outcomes from previous work on extrusion-based AM of thermosets suggested that the viscosity range obtained for resin formulations containing at least 2 wt % FS and 2 wt % CNF could be high enough to maintain filament dimensional stability after deposition.^29,39^

### 3.2 Effect of Initiator Concentrations

A study of the effects in IOC-8 and Luperox 231 concentrations in the printing resin was conducted. It is well documented that the increase of the superacid generator and radical initiator in RICFP systems results in an increase of front velocity.^38,40,41^ It has also been shown that RICFP resins likely have a greater dependence on the IOC-8 concentration than Luperox 231 concentration.^14^

In Figure S6, it is seen that the front velocity increases with either IOC-8 or Luperox 231 concentration increases from 1 to 5 phr for the printing resin consisting of 60 wt % BADGE, 20 wt % CE, and 20 wt % TEGDVE. Like the studies of increasing filler content, carbon nanofiber formulations have higher front velocities.

The greater dependency of the system on the IOC-8 concentration previously observed is not clearly seen here. Looking at the milled carbon fiber system, there is a higher front velocity at 5 phr IOC-8 and 1 or 3 phr Luperox 231 than at the opposite concentrations, 1 or 3 phr IOC-8 and 5 phr Luperox 231. For the carbon nanofiber system, front velocity with 3 or 5 phr IOC-8 and 1 phr Luperox 231 is higher than 3 or 5 phr Luperox 231 and 1 phr IOC-8. However, the same trend does not continue for a comparison of 5 phr IOC-8 and 3 phr Luperox 231 versus 5 phr Luperox 231 and 3 phr IOC-8; though, the values do appear to lie within the calculated error. Overall, there is an indication of a slightly greater dependence on the IOC-8 concentration, though not as clear as previous reports.

### 3.3 Extrusion-Based Additive Manufacturing

To enable free-standing printing, the resin must be viscous enough to limit sagging as it is extruded, and the nozzle speed must be coordinated with the front speed to obtain solidification close to the tip while avoiding clogging. For the extrusion and printing studies, resin formulations containing between 2 and 4 wt % FS and CNF were investigated because they possessed suitable viscosity. Viscosity is a critical parameter in 3D printing, influencing the flow behavior of the resin during extrusion and the layering process. As observed in Figure 5, adding fillers to the resin affected its rheological properties. During initial extrusion-based AM trials, it was experimentally observed that beyond a certain concentration (>4 wt % FS and 4 wt % CNF), the high resin viscosity made it challenging to extrude and print the material with consistent flow (above approximately 288 to 5.2 Pa·s viscosity, from 1 to 100 s^–1^ shear rate). This resulted in issues such as nozzle clogging, uneven layer deposition, and poor print quality. Therefore, a range of filler weight fractions from 2 to 4 wt % was chosen for the parametric studies. Carbon nanofibers were selected over milled carbon fibers for 3D printing because they increased front velocity more, which is preferable to lower the overall manufacturing time. In addition, less FS was needed to reach a suitable viscosity than formulations with MCF.

#### 3.3.1 Parametric Study on Composite Formulations

**Figure 6:**
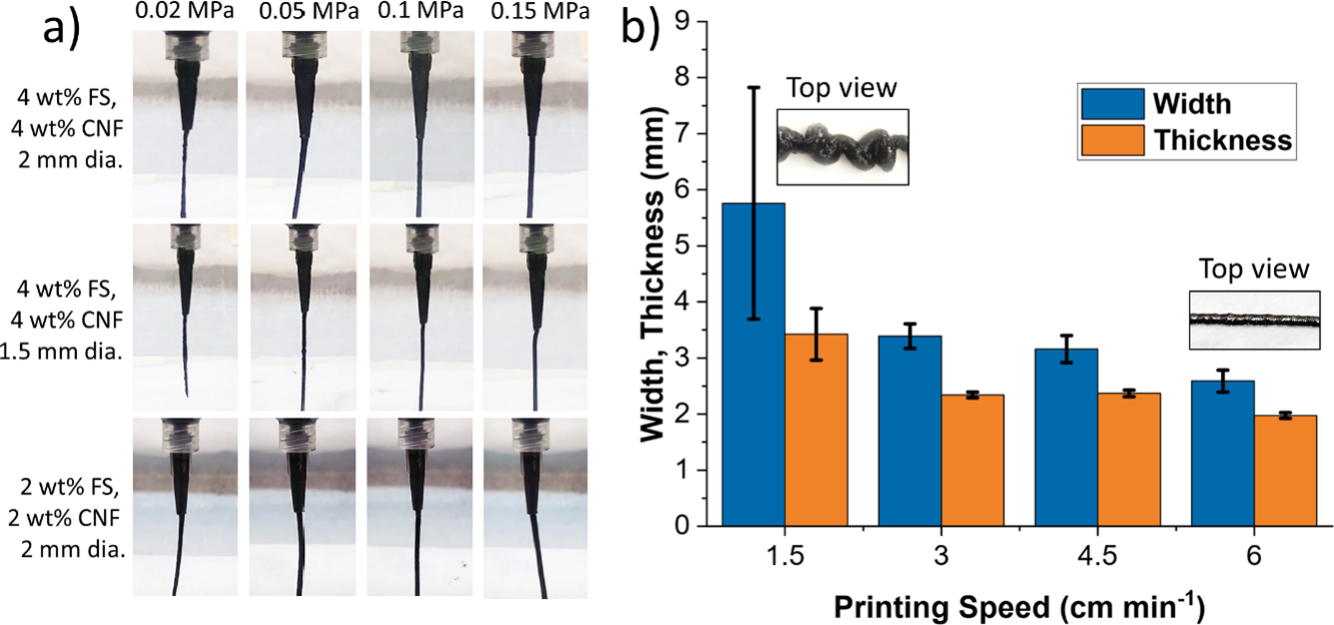
(a) Comparison of extruded filament at syringe tip for different pressures, nozzle diameters, and resin formulations. (b) Effect of printing speed on deposited filament width and thickness for 4 wt % FS, 4 wt % CNF formulation with 2 mm diameter nozzle and at 0.02 MPa pressure. Top views of the deposited filaments are shown in inset in (b) for low and high printing speeds.

Figure 6 shows a summary of preliminary extrusion experiments to find suitable pressure and printing speed ranges for extrusion of resins containing FS and CNF. Figure 6a compares extruded filaments under pressures ranging from 0.02 to 0.15 MPa for two nozzle diameters and two resin formulations. The filament exhibited smoother and more consistent behavior as the pressure and nozzle diameter increased. While the extrusion was consistent for the lowest FS and CNF weight fractions (2 wt %), it was observed that the viscosity was too low to ensure the filament would retain its shape after extrusion and deposition (a range from approximately 1.7 to 63 Pa·s, depending on shear rate, as indicated in Figure 5). This was noted for 3 wt % formulations as well, confirming that 4 wt % would be the most suitable for free-standing 3D printing. From the rheological measurements described in Section 3.1.2, the 4 wt % formulation corresponds to a viscosity range from 5.2 to 288 Pa·s at a shear rate of 100 to 1 s^–1^. Assuming pipe flow in the nozzle and a flow rate consistent with the printing speed (1.5 to 6 cm min^–1^), the actual shear rate at the nozzle is estimated between 0.13 and 0.5 s^–1^.^29^ By fitting the data points presented in Figure 5 with a power law function, we can extrapolate the viscosity at the actual shear rates in the printing process between approximately 1790 and 540 Pa·s.

The 4 wt % resin formulation was then used to extrude single filaments under 0.02 MPa pressure at different printing speeds (from 1.5 to 6 cm min^–1^, a value close to the front velocity) to identify a suitable range for producing filaments with consistent width and thickness while matching the front velocity of the resin system. The results are presented in Figure 6b, confirming that filament width and thickness decreased with a low standard deviation as the speed increased up to 6 cm min^–1^. This indicates that using the 4 wt % resin formulation, in combination with a nozzle diameter of 2 mm and a pressure of 0.02 MPa, could be suitable for free-standing printing as it would be possible to coordinate front and printing speeds. Using a higher pressure would require a higher printing speed to maintain consistent filament extrusion but could exceed the front velocity, leading to filament sagging and unsuccessful free-standing printing.

#### 3.3.2 Mechanical Characterization of Composite Specimens

**Figure 7:**
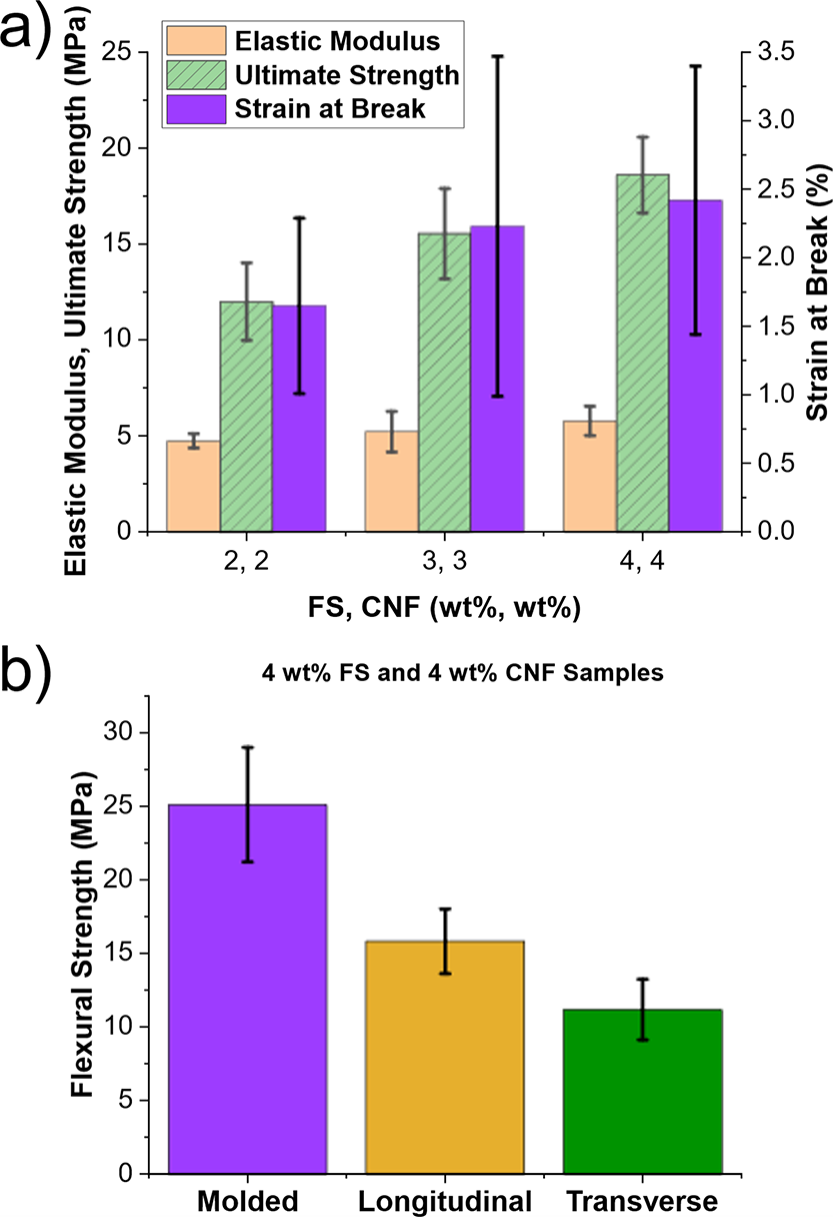
(a) Comparison of tensile properties for as-molded FP resin specimens with increasing FS and CNF percentage and (b) comparison of flexural strength for FP resin specimens (4 wt % FS, 4 wt % CNF) as-molded and 3D-printed in the longitudinal and transverse directions.

**Figure 8:**
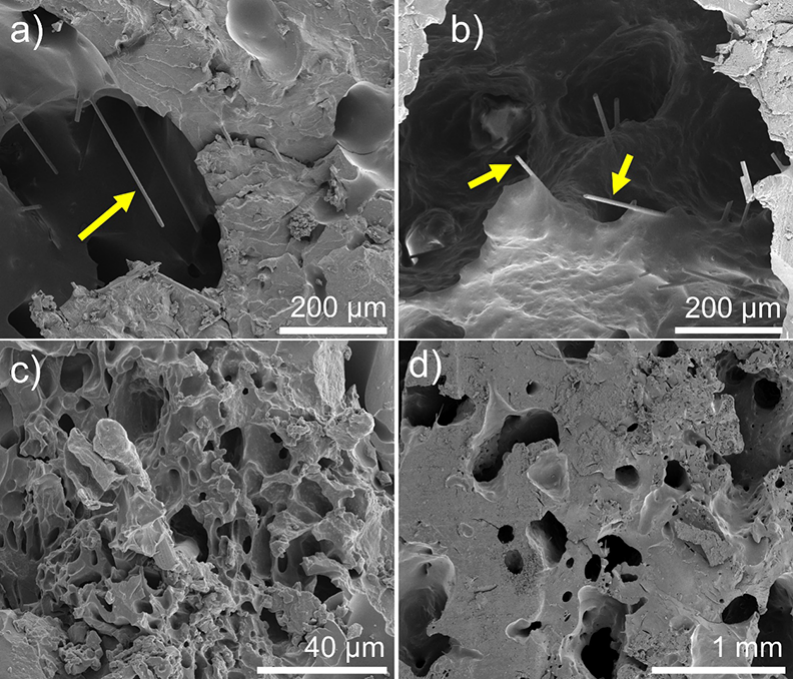
SEM images of composites produced by RICFP with 4 wt % milled carbon fibers and 7 wt % FS. (a) and (b) show detail of the milled carbon fibers, while (c) and (d) show detail of the polymer morphology. Scale bars and arrows pointing to the fibers are shown in each image.

**Figure 9:**
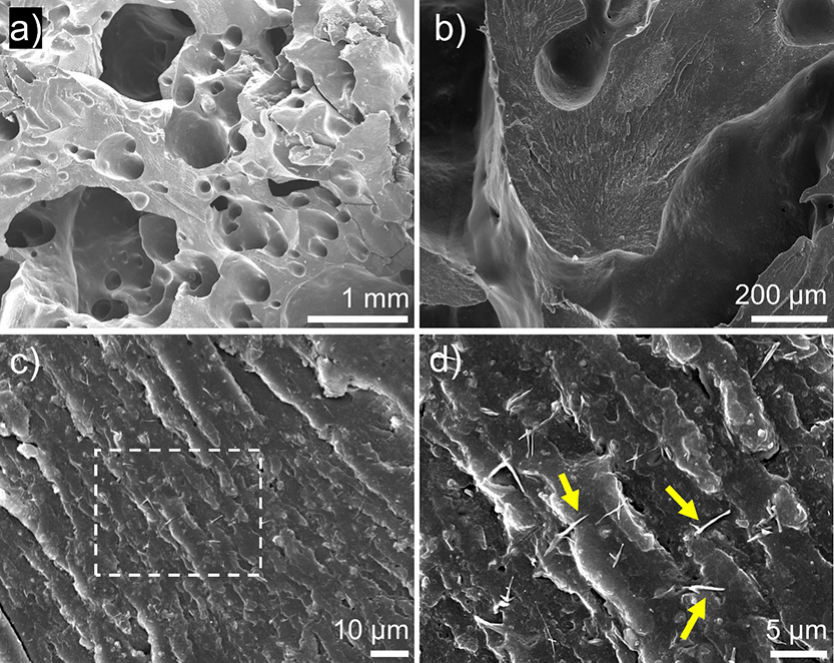
SEM images of composites produced by RICFP with 4 wt % FS and 4 wt % CNF, where (a) and (b) show detail of the polymer morphology and (d) shows detail of the CNFs in the zoomed-in region of (c), as indicated by the dashed rectangle. Scale bars are included in all images. Arrows pointing to carbon nanofibers are shown in (d).

As discussed in Section 3.1.1, CE was added to the TEGDVE-epoxy formulation to improve rigidity while maintaining reactivity and supporting the front propagation. The effect of FS and CNF weight fractions on the tensile properties of the resin system, for as-molded specimens, is shown in Figure 7a. Representative stress–strain curves for the tensile tests are shown in Figure S7. The average ultimate strength increased with filler weight fraction, indicating effective reinforcement of the specimens. Similarly, the elastic modulus showed a slight increase as the filler loading increased from 2 to 4 wt %. The strain at break confirmed the rigid, brittle behavior of the resin system at all weight fractions. However, the large standard deviations for strain at break values imply that the difference between weight fractions is not significant. The variations between specimens were likely caused by the porous nature of the resin system after frontal polymerization. Void formation in RICFP systems has been previously reported and is caused by the decomposition of the initiators that produce gas.^40^ Representative SEM images of the MCF composites (Figure 8) show details of the composite morphology, with the presence of several voids (Figure 8c,d). This morphology was observed for both milled carbon fiber and carbon nanofiber composites. SEM images in Figure 9 show a generally uniform CNF dispersion without large agglomerates. Voids were present on the surface of the specimens as well, potentially creating damage initiation sites. Overall, it is expected that variations in tensile properties mostly depend on the presence of voids. In the literature, tensile properties of RICFP-cured epoxies without and with different fillers (woven carbon fiber plies,^34^ multiwalled carbon nanotubes (MWCNTs),^23^ or continuous carbon fibers^22^) were reported. Printed composite specimens containing 1 wt % MWCNT exhibited tensile strength in the same order of magnitude as formulations in Figure 7a.^22^ Reinforcing material and architecture (woven, continuous carbon fibers) significantly increased elastic modulus and tensile strength,^22,23,34^ but generally, comparable or higher front velocities were obtained with our 4 wt % formulation (above 5 cm min^–1^).

The influence of the manufacturing approach on the flexural behavior of composite specimens was assessed through 3PB. Figure 7b compares the flexural strength of molded specimens and 3D-printed specimens in the longitudinal and transverse directions. It shows that molded specimens possessed the highest strength, followed by 3D-printed specimens in the longitudinal and then transverse directions. It is expected that the molded specimens displayed the highest strength because the fabrication process involved the compression of the specimen between two acrylic sheets. This created more even front propagation and surfaces compared to 3D-printed specimens, for which the bottom surface in contact with the printing bed exhibited a more porous morphology. The reduced strength of 3D-printed specimens may be further attributed to adhesion issues between adjacent filaments and possible defects introduced by voids, especially for those printed in the transverse direction. The lowest strength in the transverse direction could also be explained by preferential CNF alignment along the extruded filaments, leading to lower flexural properties compared to the longitudinal direction, as suggested in the literature for different frontal polymerization systems.^20^

The void content of the polymers produced by frontal polymerization was estimated using gravimetric density measurements based on ASTM D2734, with limitations of sample size and a slight modification of the provided equation to replace the theoretical composite density with a measured composite density of a minimal void sample. Comparing densities of oven-cured samples with minimal voids to the densities of samples produced by frontal polymerization with many voids, it was found that samples with 4 wt % CNF and 4 wt % FS had an average 41 ± 4.1% void content, while samples with 4 wt % MCF and 7 wt % FS had an average 58 ± 5.9% void content. Both results indicate that there is a high number of voids in the polymer, which can explain some of the variability in the measured mechanical performance. There was some difficulty producing a reference sample to obtain the density without voids present. Future investigations of the void content and methods to reduce it are recommended to use microcomputed tomography for porosity analysis.

Voids could be reduced by further optimization of initiator concentration in these systems since the decomposition of the initiators is the biggest contributor to the issue. Another potential solution could be the introduction of fillers to act as nucleation sites for bubbles and generate a more uniform void content. Benzopinacol could also be used as a thermal radical initiator, which has been shown to produce no gas,^16^ but can be difficult to dissolve in these epoxy-vinyl ether resins, requiring solvents that can negatively impact front propagation.^23,25^ Luperox 231 does not have this issue of solubility as it is a liquid peroxide.

#### 3.3.3 Free-Standing Additive Manufacturing Demonstration

**Figure 10:**
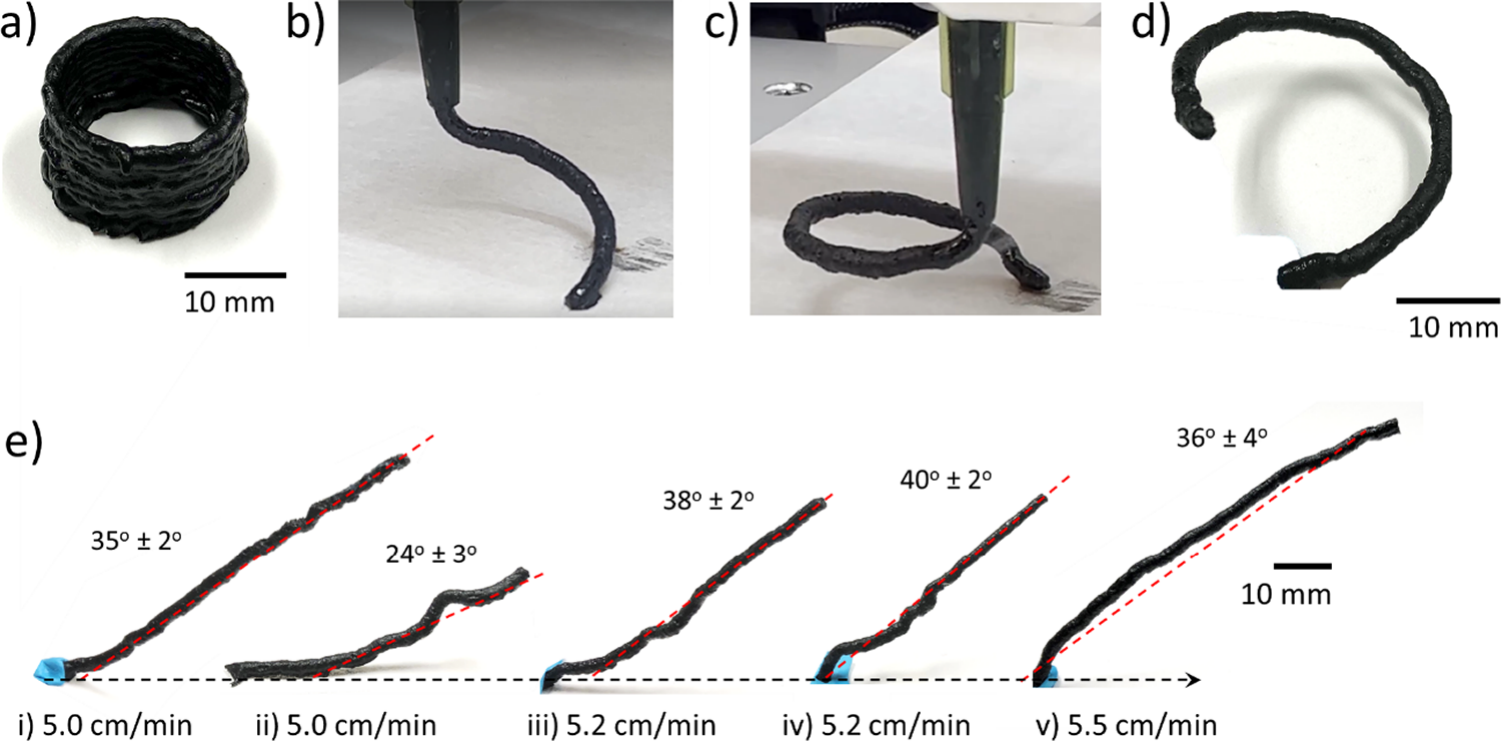
(a) Cylindrical specimen printed with robotic AM system, (b) and (c) show examples of free-standing helical filaments during extrusion, (d) helical specimen from (c) after extrusion, and (e) free-standing filaments at a 40° angle at different printing speeds and reaction initiation delays.

Figure 10 shows different 3D printing geometries to assess the feasibility of printing approaches. Layer-by-layer cylindrical geometries were first 3D-printed with a robotic AM system (Figure 10a) to assess the main issues. Initially, coordination between the front velocity and extruder speed was attempted by selecting a printing speed of 6 cm min^–1^. However, as layers were deposited on top of existing layers, which remained at high temperature, the front propagated through the syringe tip, clogging the extruder. To avoid this issue, a higher printing speed between 40 and 45 cm min^–1^ was used, which allowed successful fabrication of the planar specimen.

For free-standing printing, the main challenge was to achieve solidification of the filament as it is extruded by maintaining an adequate distance between the nozzle and front. A print speed that closely matches the front velocity is optimal for this process. Several trials were performed to study the effect of reaction initiation with a soldering iron and the printing speed. Single filaments were extruded at a 40° angle at printing speeds between 5.0 and 5.5 cm min^–1^, as shown in Figure 10e. Figure 10e-i,ii compares different reaction initiation delays at the same printing speed. A longer reaction initiation delay, where the distance between the nozzle and the front was above 10 mm, led to a specimen geometry and angle significantly deviating from the planned path at 40°. A printing speed of 5.2 cm min^–1^ (Figure 10e-iii,iv) with a reaction initiation delay between approximately 5 and 7 mm allowed free-standing printing of filaments with angles between 38° ± 2° and 40° ± 2°. However, as the reaction initiation method required contact with the filament upon extrusion, the base of the filaments was inconsistent. Finally, a higher printing speed of 5.5 cm min^–1^, along with a reaction initiation delay lower than 5 mm, showed a curved filament shape. This indicates the reaction was initially well-coordinated with the nozzle, but the distance between the nozzle and front increased over time due to the printing speed. From those initial trials, a printing speed of 5.2 cm min^–1^ was selected, and the reaction initiation method was improved by using two UV spotlights to eliminate physical contact. Helical geometries were then manufactured to demonstrate the feasibility of free-standing 3D printing with the proposed resin system (Figure 10b–d). Future work should address void formation during frontal polymerization to create structures with higher dimensional stability. Depending on the printed geometry, print speed must be adjusted to prevent front propagation between layers, which would lead to failure of the print. This is shown by the significant differences between the print speeds of free-standing helical (Figure 10b–d) and layered cylindrical (Figure 10a) structures.

## 4 Conclusions

In this work, the optimization and application of a formulation that uses RICFP to 3D print free-standing structures was studied. During the process of optimization, it was found that increasing the ratio of tri(ethylene glycol) divinyl ether to BADGE increased the front velocity substantially. However, with this increasing ratio, the polymer became more flexible than the stiff polymer that was desired. To balance the reactivity and physical properties, a cycloaliphatic epoxy was added. The effect of the initiator concentration was studied and shown to have a slightly greater dependence on the IOC-8 concentration than the Luperox 231 concentration. The addition of filler can increase the viscosity and affect front kinetics, as seen with the addition of milled carbon fibers and carbon nanofibers, which are both thermally conductive and have high thermal diffusivity. Carbon nanofibers were found to affect the front velocity more than milled carbon fibers, which is likely due to the higher surface area of carbon nanofibers, differences in thermal conductivity of the fillers, or the lower loadings of the fillers compared to previous reports in the literature. Interestingly, for both fillers, the front velocity was higher in a 1.5 mm layer rather than a 6 mm layer.

To demonstrate extrusion-based additive manufacturing, a desktop 3D printer was modified to control resin extrusion and deposition using a digital syringe dispenser. A parametric study compared the effect of air pressure, nozzle diameter, and filler weight fraction on extrusion behavior, revealing that a formulation containing 4 wt % FS and 4 wt % carbon nanofibers was the most suitable for free-standing printing. The main goal was to achieve a viscosity for consistent extrusion through the nozzle while avoiding sagging of the extruded material at the nozzle tip. An air pressure of 0.02 MPa allowed the extrusion of dimensionally stable filaments at a printing speed matching the front velocity (between 5 and 6 cm min^–1^). Flexural properties of molded and 3D-printed specimens were obtained through 3PB tests, showing that specimens printed in the transverse direction exhibited the lowest strength. This is likely due to the presence of voids within and between filaments, adhesion issues, and preferential carbon nanofiber alignment along the filaments. SEM confirmed the porous morphology, resulting from the decomposition of the initiators, producing gas during frontal polymerization. Determination of void content by density measurements showed a high average void content (41 ± 4.1% for CNF samples and 58 ± 5.9% for MCF samples). Finally, free-standing printing was successfully demonstrated with single, angled filaments and helical geometries. Future work should focus on reduction of void formation, which would improve the mechanical performance of 3D-printed specimens. In addition, a more detailed study is needed to investigate the causes of increased front velocities at thinner layers with carbon nanofibers and milled carbon fibers and the interactions of the carbon-based fillers with the resin system.

## Supporting Information Available

Mechanism of radical-induced cationic frontal polymerization, differential scanning calorimetry curves for the system containing the printing resin, effects of initiator concentration on front velocity, and representative stress–strain curves obtained from tensile testing of the molded frontal polymerization specimens (PDF)

## References

[ref1] Suslick B. A.; Hemmer J.; Groce B. R.; Stawiasz K. J.; Geubelle P. H.; Malucelli G.; Mariani A.; Moore J. S.; Pojman J. A.; Sottos N. R. Frontal Polymerizations: From Chemical Perspectives to Macroscopic Properties and Applications. Chem. Rev. 2023, 123 (6), 3237–3298. 10.1021/acs.chemrev.2c00686. 36827528 PMC10037337

[ref2] Pojman J. A. Frontal Polymerization. In Polymer Science: A Comprehensive Reference, Matyjaszewski K.; Möller M. Eds.; Elsevier BV, 2012; 4, 957–980.

[ref3] Chechilo N. M.; Enikolopyan N. S. Structure of the Polymerization Wave Front and Propagation Mechanism of the Polymerization Reaction. Dokl. Phys. Chem. 1974, 214 (5), 174–176.

[ref4] Chechilo N. M.; Khvilivitskii R. J.; Enikolopyan N. S. On the Phenomenon of Polymerization Reaction Spreading. Dokl. Akad. Nauk. SSSR 1972, 204 (N5), 1180–1181.

[ref5] Bynum S.; Tullier M.; Morejon-Garcia C.; Guidry J.; Runnoe E.; Pojman J. A. The effect of acrylate functionality on frontal polymerization velocity and temperature. J. Poly. Sci: Part A: Pol. Chem. 2019, 57 (9), 982–988. 10.1002/pola.29352.

[ref6] Pojman J. A. Traveling Fronts of Methacrylic Acid Polymerization. J. Am. Chem. Soc. 1991, 113, 6284–6286. 10.1021/ja00016a063.

[ref7] Gary D. P.; Bynum S.; Thompson B. D.; Groce B. R.; Sagona A.; Hoffman I. M.; Morejon-Garcia C.; Weber C.; Pojman J. A. Thermal transport and chemical effects of fillers on free-radical frontal polymerization. J. Polym. Sci. 2020, 58 (16), 2267–2277. 10.1002/pol.20200323.

[ref8] Mariani A.; Fiori S.; Chekanov Y.; Pojman J. A. Frontal Ring-Opening Metathesis Polymerization of Dicyclopentadiene. Macromolecules 2001, 34, 6539–6541. 10.1021/ma0106999.

[ref9] Stawiasz K. J.; Wendell C. I.; Suslick B. A.; Moore J. S. Photoredox-Initiated Frontal Ring-Opening Metathesis Polymerization. ACS Macro Lett. 2022, 11, 780–784. 10.1021/acsmacrolett.2c00248. 35638608

[ref10] Suslick B. A.; Stawiasz K. J.; Paul J. E.; Sottos N. R.; Moore J. S. Survey of Catalysts for Frontal Ring-Opening Metathesis Polymerization. Macromolecules 2021, 54 (11), 5117–5123. 10.1021/acs.macromol.1c00566.

[ref11] Chen S. H.; Sui J.; Chen L. Segmented polyurethane synthesized by frontal polymerization. Colloid Polym. Sci. 2005, 283, 932–936. 10.1007/s00396-004-1262-5.

[ref12] Fiori S.; Mariani A.; Ricco L.; Russo S. First Synthesis of a Polyurethane by Frontal Polymerization. Macromolecules 2003, 36, 2674–2679. 10.1021/ma0211941.

[ref13] Mariani A.; Bidali S.; Fiori S.; Sangermano M.; Malucelli G.; Bongiovanni R.; Priola A. UV-Ignited Frontal Polymerization of an Epoxy Resin. J. Poly. Sci. Part A Polym. Chem. 2004, 42, 2066–2072. 10.1002/pola.20051.

[ref14] Groce B. R.; Gary D. P.; Cantrell J. K.; Pojman J. A. Front velocity dependence on vinyl ether and initiator concentration in radical-induced cationic frontal polymerization of epoxies. J. Polym. Sci. 2021, 59 (15), 1678–1685. 10.1002/pol.20210183.

[ref15] Malik M. S.; Schlogl S.; Wolfahrt M.; Sangermano M. Review on UV-Induced Cationic Frontal Polymerization of Epoxy Monomers. Polym. 2020, 12 (9), 2146 10.3390/polym12092146. PMC7570253 32962306

[ref16] Bomze D.; Knaack P.; Liska R. Successful radical induced cationic frontal polymerization of epoxy-based monomers by C-C labile compounds. Polym. Chem. 2015, 6, 8161–8167. 10.1039/C5PY01451D.

[ref17] Groce B. R.; Lane E. E.; Gary D. P.; Ngo D. T.; Ngo D. T.; Shaon F.; Belgodere J. A.; Pojman J. A. Kinetic and Chemical Effects of Clays and Other Fillers in the Preparation of Epoxy–Vinyl Ether Composites Using Radical-Induced Cationic Frontal Polymerization. ACS Appl. Mater. Interfaces 2023, 15, 19403–19413. 10.1021/acsami.3c00187. 37027250 PMC10119861

[ref18] Robertson I. D.; Yourdkhani M.; Centellas P. J.; Aw J. E.; Ivanoff D. G.; Goli E.; Lloyd E. M.; Dean L. M.; Sottos N. R.; Geubelle P. H.; Moore J. S.; White S. R. Rapid energy-efficient manufacturing of polymers and composites via frontal polymerization. Nature 2018, 557 (7704), 223–227. 10.1038/s41586-018-0054-x. 29743687

[ref19] Ziaee M.; Naseri I.; Johnson J. W.; Franklin K. A.; Yourdkhani M. Frontal Polymerization and Three-Dimensional Printing of Thermoset Polymers with Tunable Thermomechanical Properties. ACS Appl. Polym. Mater. 2023, 5 (3), 1715–1724. 10.1021/acsapm.2c01736.

[ref20] Ziaee M.; Johnson J. W.; Yourdkhani M. 3D Printing of Short-Carbon-Fiber-Reinforced Thermoset Polymer Composites via Frontal Polymerization. ACS Appl. Mater. Interfaces 2022, 14 (14), 16694–16702. 10.1021/acsami.2c02076. 35353492

[ref21] Zhao S.; Li J.; An M.; Jin P.; Zhang X.; Luo Y. Energy-efficient manufacturing of polymers with tunable mechanical properties by frontal ring-opening metathesis polymerization. Polym. Adv. Technol. 2023, 34 (1), 441–445. 10.1002/pat.5895.

[ref22] Zhang Z.; Liu R.; Li W.; Liu Y.; Luo H.; Zeng L.; Qiu J.; Wang S. Direct writing of continuous carbon fibers/epoxy thermoset composites with high-strength and low energy-consumption. Addit. Manuf. 2021, 47, 102348 10.1016/j.addma.2021.102348.

[ref23] Zhang Z.; Gao C.; Liu R.; Qiu J.; Pei Z.; Wang S. 3D Printing of Frontal-polymerized Multiscale Epoxy Thermoset and Composites. Manuf. Lett. 2022, 33, 640–643. 10.1016/j.mfglet.2022.07.079.

[ref24] Hiremath N.; Bhat G. High-performance carbon nanofibers and nanotubes. In Structure and Properties of High-Performance Fibers, Bhat G. Ed.; Woodhead Publishing, 2017; 79-109.

[ref25] Zhang Z.; Gao C.; Liu R.; Li W.; Qiu J.; Wang S. Catalyzed frontal polymerization-aided 3D printing of epoxy thermosets. Additive Manufacturing Letters 2022, 2, 100030 10.1016/j.addlet.2022.100030.

[ref26] Gao C.; Liu R.; Li W.; Qiu J.; Wang S. Collaborative printing and in-situ frontal curing of highly-viscous thermosetting composites. Journal of Manufacturing Processes 2023, 89, 1–9. 10.1016/j.jmapro.2023.01.048.

[ref27] Yoong Ahm Kim T. H.; Endo M.; Dresselhaus M. S.; Carbon Nanofibers. In Springer Handbook of Nanomaterials, Vajtai R. Ed.; Springer-Verlag: Berlin Heidelberg, 2013.

[ref28] Velazquez L.; Palardy G.; Barbalata C. Design and integration of end-effector for 3D printing of novel UV-curable shape memory polymers with a collaborative robotic system. arXiv 2021, 10.48550/arXiv.2108.10810.

[ref29] Velazquez L.; Palardy G.; Barbalata C. A robotic 3D printer for UV-curable thermosets: dimensionality prediction using a data-driven approach. International Journal of Computer-Integrated Manufacturing 2023, 1 10.1080/0951192X.2023.2257652.

[ref30] Malik M. S.; Wolfahrt M.; Sangermano M.; Schlögl S. Effect of a Dicycloaliphatic Epoxide on the Thermo-Mechanical Properties of Alkyl, Aryl Epoxide Monomers Cured via UV-Induced Cationic Frontal Polymerization. Macromol. Mater. Eng. 2022, 307 (7), 2100976 10.1002/mame.202100976.

[ref31] Gao Y.; Shaon F.; Kumar A.; Bynum S.; Gary D.; Sharp D.; Pojman J. A.; Geubelle P. H. Rapid frontal polymerization achieved with thermally conductive metal strips. Chaos 2021, 31 (7), 073113 10.1063/5.0052821. 34340327

[ref32] Gao Y.; Li S.; Kim J.-Y.; Hoffman I.; Vyas S. K.; Pojman J. A.; Geubelle P. H. Anisotropic frontal polymerization in a model resin–copper composite. Chaos 2022, 32 (1), 013109 10.1063/5.0077552. 35105137

[ref33] Goli E.; Parikh N. A.; Yourdkhani M.; Hibbard N. G.; Moore J. S.; Sottos N. R.; Geubelle P. H. Frontal polymerization of unidirectional carbon-fiber-reinforced composites. Composites, Part A 2020, 130, 105689 10.1016/j.compositesa.2019.105689.

[ref34] Sangermano M.; Antonazzo I.; Sisca L.; Carello M. Photoinduced cationic frontal polymerization of epoxy–carbon fibre composites. Polym. Int. 2019, 68 (10), 1662–1665. 10.1002/pi.5875.

[ref35] Bansal K.; Pojman J. A.; Webster D.; Quadir M. Frontal Polymerization of a Thin Film on a Wood Substrate. ACS Macro Lett. 2020, 9, 169–173. 10.1021/acsmacrolett.9b00887. 35638678

[ref36] Gao Y.; Paul J. E.; Chen M.; Hong L.; Chamorro L. P.; Sottos N. R.; Geubelle P. H. Buoyancy-Induced Convection Driven by Frontal Polymerization. Phys. Rev. Lett. 2023, 130 (2), 028101 10.1103/PhysRevLett.130.028101. 36706389

[ref37] Gao Y.; Rodriguez Koett L. E.; Hemmer J.; Gai T.; Parikh N. A.; Sottos N. R.; Geubelle P. H. Frontal Polymerization of Thin Layers on a Thermally Insulating Substrate. ACS Appl. Polym. Mater. 2022, 4, 4919–4927. 10.1021/acsapm.2c00497.

[ref38] Knaack P.; Klikovits N.; Tran A. D.; Bomze D.; Liska R. Radical induced cationic frontal polymerization in thin layers. J. Polym. Sci. Part A: Polym Chem. 2019, 57 (11), 1155–1159. 10.1002/pola.29375.

[ref39] Wang B.; Arias K. F.; Zhang Z.; Liu Y.; Jiang Z.; Sue H.-J.; Currie-Gregg N.; Bouslog S.; Pei Z.; Wang S. 3D printing of in-situ curing thermally insulated thermosets. Manuf. Lett. 2019, 21, 1–6. 10.1016/j.mfglet.2019.06.001.

[ref40] Ma Y.; Liu Z.; Qian X.; Zhao Y.; Li M.; Li P. Effect of Excessive Iodonium Salts on the Properties of Radical-Induced Cationic Frontal Polymerization (RICFP) of Epoxy Resin. Ind. Eng. Chem. Res. 2023, 62 (12), 4896–4904. 10.1021/acs.iecr.2c04134.

[ref41] Taschner R.; Liska R.; Knaack P. Iodonium Borate Initiators for Cationic Photopolymerization and Their Application in Radical-Induced Cationic Frontal Polymerization. ACS Appl. Polym. Mater. 2022, 4 (10), 7878–7890. 10.1021/acsapm.2c01465.

