# Supplementary material for: Free-Standing 3D Printing of Epoxy-Vinyl Ether Structures Using Radical-Induced Cationic Frontal Polymerization
Source: ACS Appl Polym Mater. 2023 Dec 15;6(1):572–82. doi: 10.1021/acsapm.3c02226 (PMC10788858; doi:10.1021/acsapm.3c02226)
Supplement: Supplementary file 1 — ap3c02226_si_001.pdf [file ap3c02226_si_001.pdf]

Supporting Information for

Free-Standing 3D Printing of Epoxy-Vinyl Ether Structures using Radical-Induced Cationic Frontal Polymerization

Brecklyn R. Groce[1], Alexandra V. Aucoin[1], Md Asmat Ullah[2], Jake DiCesare[1], Claire Wingfield[1], Jonathan Sardin[2], Jackson T. Harris[2], John C. Nguyen[2], Patrick Raley[2], Svetlana S. Stanley[2], Genevieve Palardy[2], and John A. Pojman[1]

[1]Department of Chemistry, Louisiana State University

Baton Rouge, LA 70803, United States

[2]Department of Mechanical and Industrial Engineering, Louisiana State University

3261 Patrick F. Taylor Hall

Baton Rouge, LA 70803, United States

* Corresponding author: gpalardy@lsu.edu

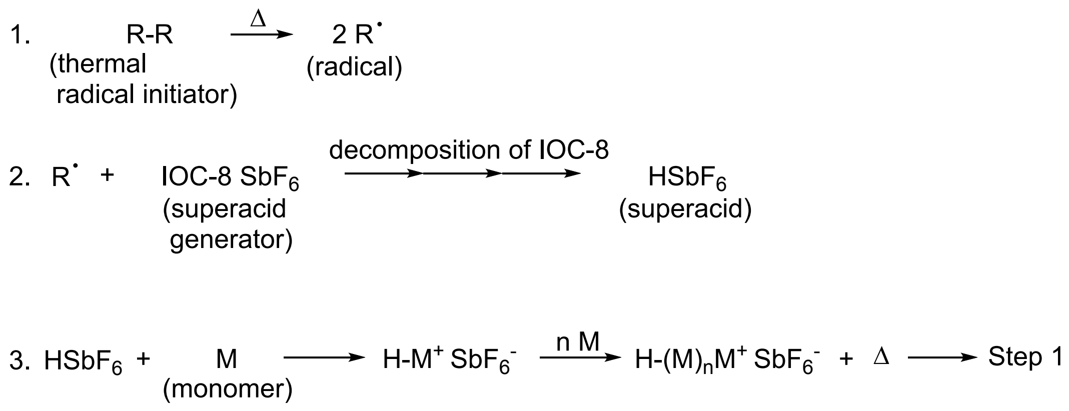

Figure S1. Mechanism of radical-induced cationic frontal polymerization.

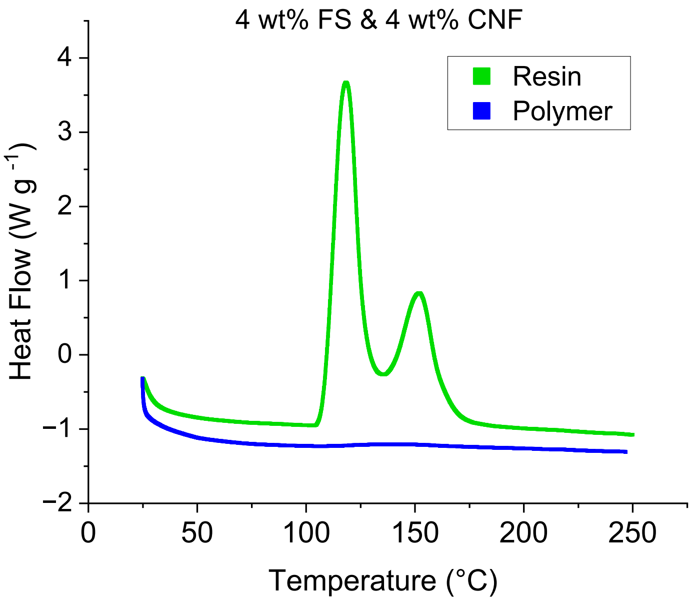

Figure S2. DSC curves for the system containing the printing resin with 4 wt% FS and 4 wt% CNF. Curves for both the uncured resin and the polymer produced by FP are shown. The procedure was a ramp rate from 25 °C to 250 °C at 10 °C min^{-1}.

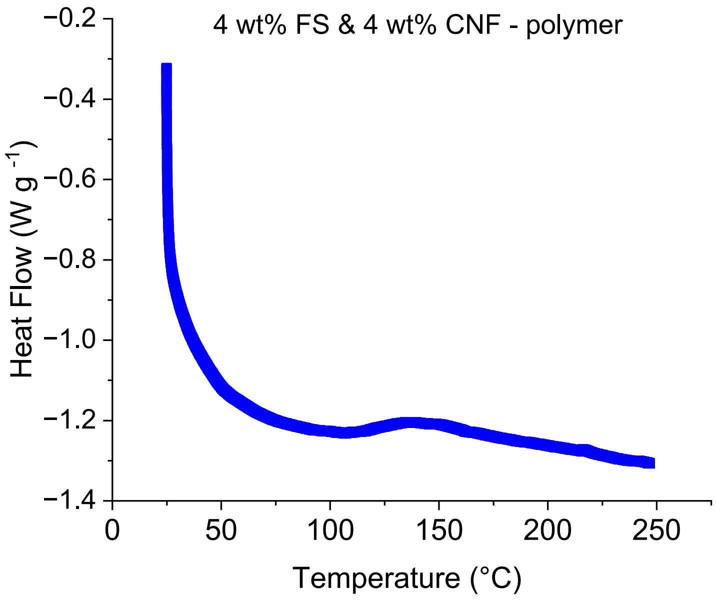

Figure S3. DSC curves for the polymer sample of system containing the printing resin with 4 wt% FS and 4 wt% CNF. The procedure was a ramp rate from 25 °C to 250 °C at 10 °C min^{-1}.

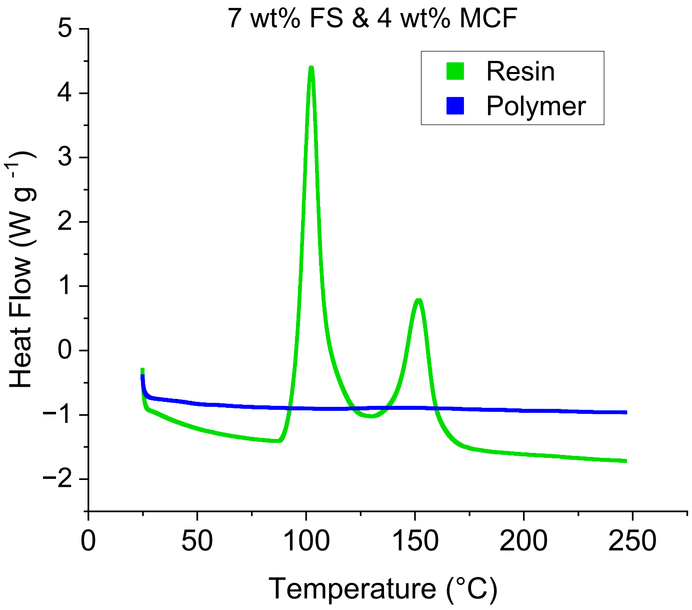

Figure S4. DSC curves for the system containing the printing resin with 7 wt% FS and 4 wt% MCF. Curves for both the uncured resin and the polymer produced by FP are shown. The procedure was a ramp rate from 25 °C to 250 °C at 10 °C min^{-1}.

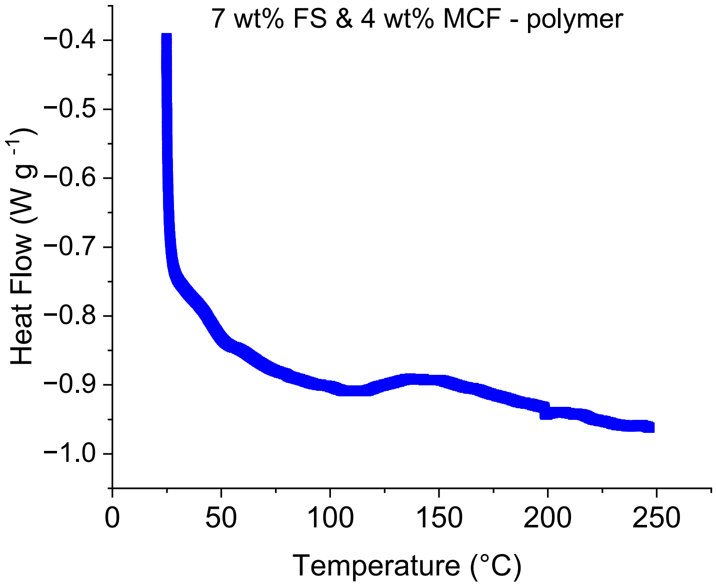

Figure S5. DSC curve for the polymer sample of system containing the printing resin with 7 wt% FS and 4 wt% MCF. The procedure was a ramp rate from 25 °C to 250 °C at 10 °C min^{-1}.

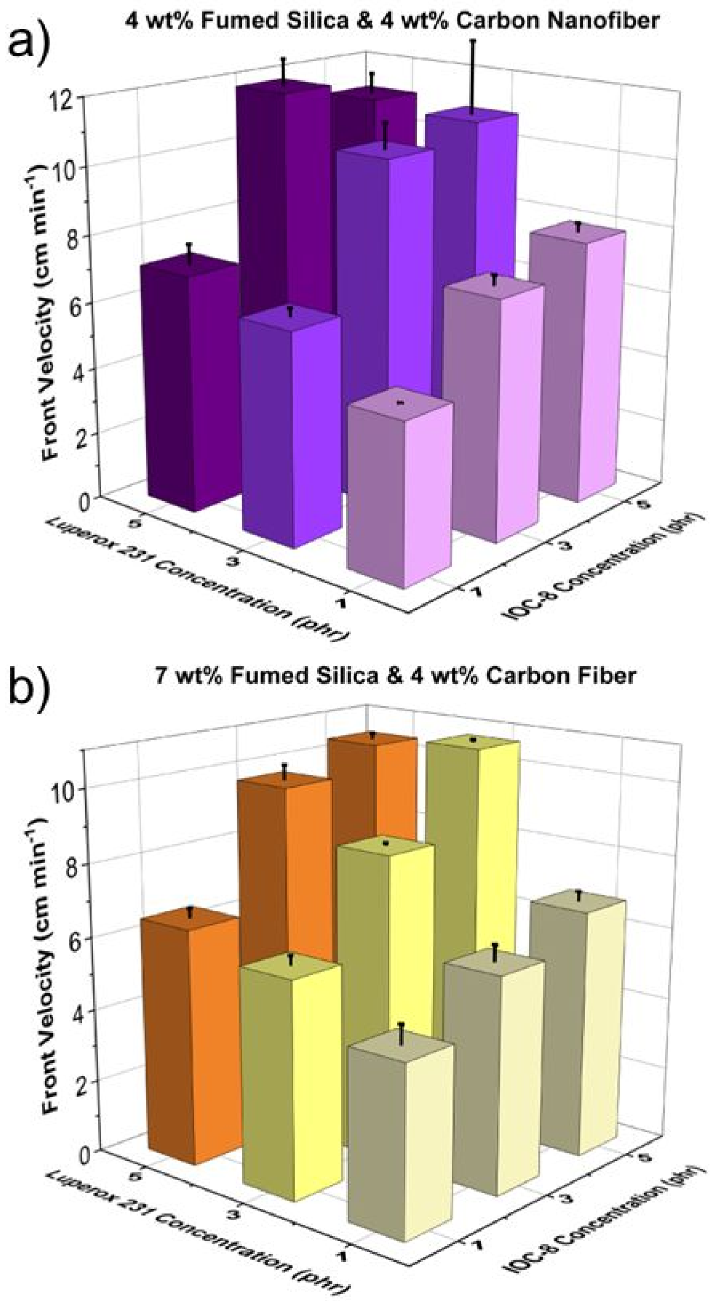

Figure S6. Effects of initiator concentration on front velocity. (a) Front velocity versus IOC-8 and Luperox 231 concentration with a resin containing 4 wt% fumed silica and 4 wt% carbon nanofiber. (b) Front velocity versus IOC-8 and Luperox 231 concentration with a resin containing 7 wt% fumed silica and 4 wt% milled carbon fiber.

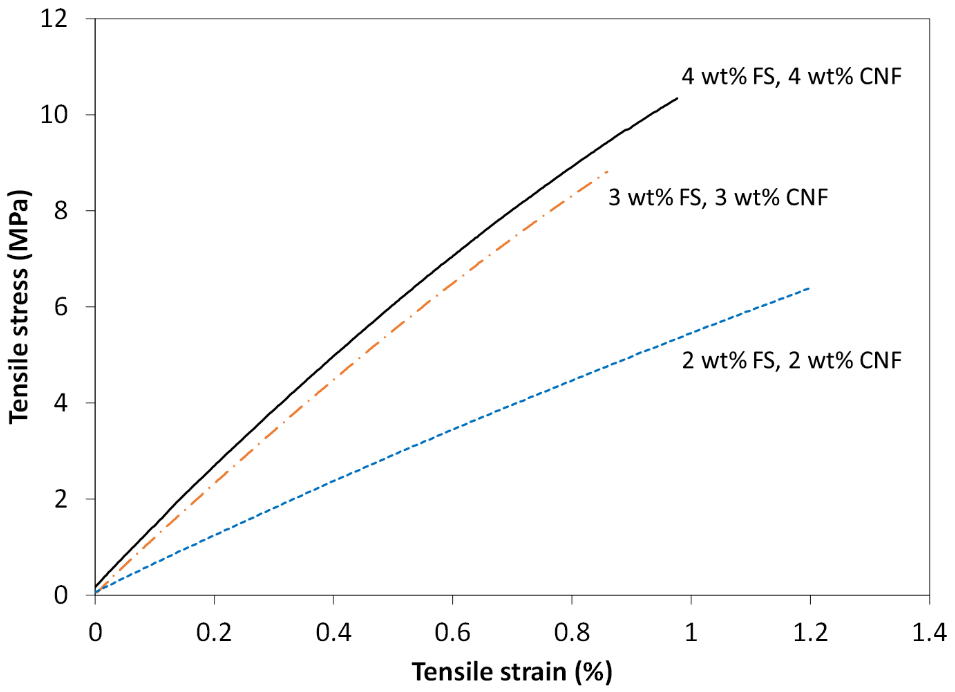

Figure S7. Representative stress-strain curves obtained from tensile testing of the molded FP specimens with increasing FS and CNF percentages.
